# Quality of life utility values for hereditary haemochromatosis in Australia

**DOI:** 10.1186/s12955-016-0431-9

**Published:** 2016-02-29

**Authors:** Barbara de Graaff, Amanda Neil, Kristy Sanderson, Kwang Chien Yee, Andrew J. Palmer

**Affiliations:** Menzies Institute for Medical Research, University of Tasmania, Medical Sciences Building 1, 17 Liverpool St, Private Bag 23, Hobart, TAS 7000 Australia; School of Medicine, University of Tasmania, Medical Sciences Building 2, 17 Liverpool St, Private Bag 68, Hobart, TAS 7000 Australia

## Abstract

**Background:**

Hereditary hemochromatosis (HH) is a common autosomal recessive disorder amongst persons of northern European heritage. If untreated, iron accumulates in parenchymal tissues causing morbidity and mortality. As diagnosis often follows irreversible organ damage, screening programs have been suggested to increase early diagnosis. A lack of economic evidence has been cited as a barrier to establishing such a program. Previous analyses used poorly estimated utility values. This study sought to measure utilities directly from people with HH in Australia.

**Methods:**

Volunteers with HH were recruited to complete a web-based survey. Utility was assessed using the Assessment of Quality of Life 4D (AQOL-4D) instrument. Severity of HH was graded into four categories. Multivariable regression analysis was performed to identify parameters associated with HSUV.

**Results:**

Between November 2013 and November 2014, 221 people completed the survey. Increasing severity of HH was negatively associated with utility. Mean (standard deviation) utilities were 0.76 (0.21), 0.81 (0.18), 0.60 (0.27), and 0.50 (0.27) for categories 1–4 HH respectively. Lower mean utility was found for symptomatic participants (categories 3 and 4) compared with asymptomatic participants (0.583 v. 0.796). Self-reported HH-related symptoms were negatively associated with HSUV (r = −0.685).

**Conclusions:**

Symptomatic stages of HH and presence of multiple self-reported symptoms were associated with decreasing utility. Previous economic analyses have used higher utilities which likely resulted in underestimates of the cost effectiveness of HH interventions. The utilities reported in this paper are the most robust available, and will contribute to improving the validity of future economic models for HH.

## Background

Hereditary hemochromatosis (HH) is a common autosomal recessive disorder in populations of northern European heritage [1, 2]. It is characterised by increased iron absorption caused by a defect in the HFE gene. Several mutations have been identified: C282Y, H63D and S56C [3–5]. C282Y homozygosity accounts for 80 to 90 % of people diagnosed with iron-overload, with the other mutations uncommonly associated with iron overload [6, 7]. It has been hypothesised that HH is most prevalent in northern European populations due to a mutation occurring in Central Europe, hence the description ‘Celtic mutation” [8]. Prevalence of C282Y homozygosity has been reported to be between 1 in 150 to 200 persons of Northern European ancestry. Amongst populations of different heritage, prevalence is much lower: 1 in 300 Hispanics; 1 in 1,000 Native Americans; 1 in 1,000, 000 Asians [9–13]. Whilst prevalence of other genotypes is more common (1 in 50 C282Y/H63D compound heterozygotes), the burden of disease associated with these mutations is low [4, 14].

In a proportion of C282Y homozygotes, elevated hepcidin production increases the absorption of dietary iron, which is stored in the parenchymal tissues of the heart, liver and pancreas. If left untreated, iron overload can be a cause of morbidity and mortality, including multiple arthropathies, type 2 diabetes, liver disease and heart disease [15–17]. HH and iron overload is commonly diagnosed by conducting iron studies (transferrin saturation and serum ferritin) with confirmatory genotyping. Treatment consists of regular therapeutic venesection.

Rates of clinical penetrance (i.e. expression of disease) reported in the literature vary, in part due to different definitions. Some authors have defined penetrance as irreversible organ damage, such as cirrhosis or hepatocellular carcinoma, whilst other have included a spectrum of health states, from elevated iron stores and serum iron through to irreversible organ damage. Recent studies have reported rates of cirrhosis of the liver amongst C282Y homozygotes to be between 2 and 6 % [10, 18, 19]. When penetrance is defined as elevated iron stores and serum iron through to irreversible organ damage, rates of 28.4 % for male and 1.2 % for female C282Y homozygotes have been recently reported [10].

Whilst diagnosis and prevention of iron overload in genetically susceptible patients is relatively straightforward, the non-specific nature of early symptomatology, in that this can be experienced by people with clinically normal iron levels, contributes to some patients being diagnosed only after irreversible organ damage has occurred [20–23]. Effective treatment is readily available, therefore early diagnosis and timely treatment leads to substantial improvements in patient outcomes. Population screening strategies have been proposed as an approach to increase early identification of people with HH, thereby reducing the potential burden of disease associated with iron overload [24–28].

Whilst HH is a condition that fulfils several of the criteria set out by the World Health Organisation for population screening programs [29], a lack of robust health economic data has been cited as a hurdle to implementing such a program [24, 25, 30, 31]. Considerable limitations have been identified in the economic evaluations of HH screening programs that have been published to date [32, 33].

Cost effectiveness analyses and cost utility analyses give rise to a ratio of the difference in costs and effectiveness between two or more health interventions. The cost of an intervention is measured in monetary units and effectiveness may be measured unidimensionally for cost effectiveness analyses (e.g. life years gained) or by means of a multidimensional instrument (such as the EQ-5D, SF-6, AQOL-4D) for cost utility analyses. Importantly, multi-attribute utility instruments allow for calculation of an individual’s utility (HSUV): a measure of the strength of preference for a particular health state. Utilities are measured on a scale of zero to one, with one representing full health, and zero, death. Some instruments such as the AQOL-4D and the EQ-5D allow for negative values, as certain states may be considered worse than death [36, 37]. When a utility is combined with life years gained (LYG), the outcome reflects both morbidity and mortality: quality adjusted life years (QALYs). A cost per QALY can then be reported, the preferred unit of measurement of many decision makers, such as the UK’s National Institute for Health and Care Excellence (NICE) [34] and the Australian Pharmaceutical Benefits Advisory Committee (PBAC) [35].

To date, just four cost utility studies of HH screening programs have been published [33]. The studies did not report the sources of the utilities used, and the estimates employed for conditions such as healthy state, heart disease and cirrhosis of the liver were markedly higher than reported for comparable populations [33]. Such use of elevated utility values is likely to result in underestimates of the potential gains associated with screening programs, which in turn may impact on policy decisions regarding provision of HH screening programs.

The purpose of this study was to assess the utilities for a sample of people with HH with different stages of disease severity using a multi-attribute utility instrument.

## Methods

A web-based cross-sectional study using convenience sampling was conducted across Australia. Multiple recruitment strategies were used: the national support group, Hemochromatosis Australia (HA), sent emails to all members on behalf of the researchers informing them of the project and the web address; the link to the survey was placed on HA’s website; flyers outlining the study were sent to large Australian metropolitan hepatology, haematology and gastroenterology clinics, along with general practitioners sourced from HA’s referral network; advertisements were placed on social media sites; and newspaper articles about the condition and the study were published. In addition, case finding was conducted in all Tasmanian public hospitals. All patients admitted between July 2009 and June 2014 with a diagnosis of HH, as identified in the International Statistical Classification of Diseases and Related Health Problems, Tenth Revision, Australian Modification (ICD-10-AM) by code E831: Disorders of Iron Metabolism, were sent letters by the research group, informing them of the study and inviting them to participate. Only names and postal details were supplied to the researchers. Eligibility criteria included a diagnosis of haemochromatosis, residing in Australia, aged 18 or older and provision of written informed consent. Ethical approval for the study was granted by the Tasmanian Health and Medical Research Ethics Committee (H0013564).

## Measurements

### HSUV

Utility was measured using the Assessment of Quality of Life 4D instrument (AQOL-4D) [38]. The AQOL-4D is a 12 item questionnaire that provides a global health state utility value. It consists of four separate dimensions: independent living, relationships, mental health and senses. The HSUV is scored on a scale from −0.04 to 1.00. A score of one represents optimal health, a score of zero represents a state equivalent to death, and scores less than zero represent states worse than death [38]. This instrument was chosen as it is sensitive to a broad range of conditions and health states [39], Australian population normative data were available for comparison [40], and due to cost and software limitations associated with the use of other instruments. AQOL HSUV were calculated using syntax supplied by the AQOL Group [41].

### HH-related health states

Stages of HH were categorised using the framework published by the European Association for the Study of the Liver (EASL) [16]. The expert panel identified a lack of generalizability of much of the research into HH, in part due to researchers and clinicians using different definitions or descriptions of HH, i.e. genetic mutation only, through to organ damage. To address this, EASL recommended using uniform categorisation of the different stages of HH. These categories are described in Table 1. Participants were provided with this matrix, and asked to categorise their condition. These self-categorisations were verified by cross-checking responses with regard to recent experience of HH comorbidities. Just one discrepancy was identified: recoding for the more conservative categorisation was carried out and comorbidities were assumed to be unrelated to HH.

**Table 1:** Categories of HH [11]

Category 1	Genetic mutation only (C282Y homozygotes, H63D heterozygotes and compound heterozygotes)
Category 2	Genetic mutation and elevated iron studies, either transferrin saturation or serum iron
Category 3	Genetic mutation, elevated iron levels and early symptoms, including arthritis, fatigue
Category 4	Genetic mutation, elevated iron levels and organ damage

Lists of commonly reported HH-related comorbidities and symptoms were compiled following a review of the literature. Comorbidities included osteoarthritis, liver diseases (fibrosis, cirrhosis, hepatocellular carcinoma), heart failure, cardiomyopathy, Type 2 diabetes and porphyria cutanea tarda. Participants were asked if they had been diagnosed with each condition and if it was a) related to HH, b) not related to HH, or c) unsure if related to HH. Only conditions for which the participant stated were related to HH were included in analyses. In order to capture data on possible undiagnosed comorbidities and general symptoms of iron overload, participants were asked if they had experienced a range of symptoms in the last three months that they considered were related to HH. Symptoms associated with HH included the general effects of iron overload, such as fatigue, along with symptoms of liver disease, heart failure, cardiomyopathy, arthritis, porphyria cutanea tarda and changes to the reproductive system (e.g. decreased libido).

## Statistical analyses

Statistical analyses were performed using SPSS version 20.0.0. Chi square and ANOVA were used for descriptive statistics. Differences between HH utilities and data from other population groups were analysed using T-tests and Kruskal Wallis one way analysis of variance. Linear regression was carried out to identify the association between co-morbidity count and utility. A Pearson correlation coefficient was calculated for utility values and severity of HH.

## Results

### Demographics

Two hundred and seventy participants self-completed a web-based survey between November 2013 and November 2014 as part of a national cost of illness study for HH. Two hundred and twenty one participants completed the AQOL-4D. The demographic characteristics of participants are presented in Table 2. The only notable difference between participants who completed the AQOL-4D and those who did not was that the former were more likely to be employed full time (*X*^2^ = 4.254, *p* = 0.026) (Table 2).

**Table 2:** Demographic characteristics of the sample

	AQol-4D completers	AQoL-4D non-completers	*p* value
	*n* = 221	*n* = 47	
Age, mean ± SD	52.7 ± 14.2	53.6 ± 13.2	0.694
Sex (male)	41.6 %	41.3 %	0.552
Relationship status:			
currently married/defacto	79.6 %	68.1 %	0.066
Country of birth:			
Australia	83.7 %	85.1 %	0.506
United Kingdom	9.0 %	8.5 %	0.584
Highest level of education completed^a^:			
< yr 12	24.7 %	25.0 %	0.565
certificate, Trade etc.	31.7 %	39.4 %	0.245
yr 12	10.4 %	3.0 %	0.149
university	35.7 %	33.3 %	0.476
Labour force participation:			
employed full time	32.1 %	17.0 %	0.026
employed part-time	15.4 %	14.9 %	0.568
self-employed	9.0 %	10.6 %	0.455
retired	25.3 %	19.1 %	0.242
Unemployed	5.4 %	4.3 %	0.542

^a^For this question, *n* = 33 for the non-completer group

Due to the sampling techniques, it is not possible to quantify the number of people who viewed information regarding the study, thus calculating a response rate. However, for the case finding at all Tasmanian public hospitals, a response rate of 20 % was observed (37 participants from 189 letters).

### AQOL-4D HSUV

The mean utility for all participants using the AQol-4D was 0.66 (±0.26), with a range of −0.04 to 1.00 (95 % CI 0.63–0.70) (Table 3). This was lower than the Australian population norm estimated using the AQoL-4D of 0.81 (*n* = 8839, SD = 0.22, 95 % CI 0.81–0.82) [40].

**Table 3:** Comparison of HH cohort and Australian population normative utility values [23]

Variables	Mean HSUV	95 % CI	n	Males			Females			Population norm HSUV	95 % CI
				Mean HSUV	95 % CI	n	Mean HSUV	95 % CI	n		
Age group:											
20–29	0.67	0.55–0.80	10	0.75	0.53–1.00	2	0.65	0.49–0.70	8	0.86	0.85–0.87
30–39	0.72	0.62–0.80	30	0.78	0.66–0.90	7	0.70	0.59–0.81	23	0.84	0.83–0.85
40–49	0.66	0.57–0.74	39	0.72	0.57–0.84	15	0.62	0.50–0.74	24	0.81	0.80–0.82
50–59	0.63	0.54–0.70	52	0.62	0.47–0.75	19	0.63	0.54–0.71	33	0.80	0.78–0.81
60–69	0.67	0.61–0.73	67	0.70	0.62–0.78	34	0.65	0.54–0.74	33	0.80	0.78–0.81
70–79	0.61	0.47–0.73	16	0.63	0.47–0.76	11	0.56	0.21–0.92	5	0.76	0.76–0.79
Sex											
Male	0.69	0.64–0.75	92							0.82	0.81–0.83
Female	0.64	0.60–0.69	129							0.81	0.80–0.81
All	0.66	0.63–0.70	221							0.81	0.81–0.82

Note: HSUV refers to health state utility values; 95 % CI refers to the 95 % confidence interval

Univariate analyses were carried out to examine utilities for age and sex (Table 3). This showed similar values for males (0.69) and females (0.64) (*p* = 0.163). Utilities were also examined by age deciles and sex. Whilst slightly higher mean utility values were reported for males for most age deciles, none of these differences were found to be statistically significant. Overall, utility was highest for participants aged between 30 and 39 (0.72), and lowest for those aged 70–79 (0.61).

Reporting of utility by stages of severity of HH (Table 1) can help mitigate any bias due to the sampling approach. A trend of decreasing HSUV was identified with stages three and four (Table 4). A Pearson correlation coefficient was calculated to assess the relationship between mean utility and stages of HH: a moderate negative correlation was found (r = −0.366; *p* < 0.001). Whilst lower mean HSUV were reported for female participants for each category, these differences were not significant.

**Table 4:** Mean utility values by categories of HH by sex

Categories of HH	HSUV mean	SD	n	95 % CI
All participants				
Category 1	0.76	0.21	20	0.67–0.85
Category 2	0.81	0.18	63	0.76–0.85
Category 3	0.60	0.27	115	0.55–0.66
Category 4	0.50	0.27	23	0.39–0.61
All categories	0.66	0.26	221	0.63–0.70
Males				
Category 1	0.88	0.10	6	0.78–0.98
Category 2	0.85	0.12	29	0.80–0.89
Category 3	0.59	0.28	45	0.51–0.68
Category 4	0.59	0.23	12	0.44–0.74
All categories	0.69	0.27	92	0.64–0.75
Females				
Category 1	0.71	0.24	14	0.58–0.84
Category 2	0.77	0.21	34	0.70–0.85
Category 3	0.60	0.26	70	0.54–0.66
Category 4	0.41	0.29	11	0.22–0.60
All categories	0.64	0.26	129	0.60–0.69

Note: HSUV refers to health state utility values; SD standard deviation; 95 % CI refers to the 95 % confidence interval

To investigate the impact of symptomatic HH on utility, the four categories of HH were combined into asymptomatic (categories 1 and 2), and symptomatic (categories 3 and 4) participants. Utility was significantly lower for the symptomatic group for males (0.85 v. 0.59: H = 25.36, *p* < 0.001), females (0.75 v. 0.58: H = 14.90, *p* < 0.001) and overall (0.80 v. 0.58: H = 38.79, *p* < 0.001) (Table 5).

**Table 5:** Mean utility values of symptomatic HH

Categories of HH	HSUV mean	SD	n	95 % CI	Between groups^a^
All participants					
Categories 1 & 2	0.80	0.19	83	0.76–0.84	H = 38.79, *p* < 0.001
Categories 3 & 4	0.58	0.27	138	0.54–0.63	
All categories	0.66	0.26	221	0.63–0.70	
Males					
Categories 1 & 2	0.85	0.11	35	0.82–0.89	H = 25.36, *p* < 0.001
Categories 3 & 4	0.59	0.27	57	0.52–0.67	
All categories	0.69	0.26	92	0.64–0.75	
Females					
Categories 1 &2	0.75	0.22	48	0.69–0.82	H = 14.90, *p* < 0.001
Categories 3 & 4	0.58	0.27	81	0.52–0.64	
All categories	0.64	0.64	129	0.60–0.69	

Note: HSUV refers to health state utility values; SD standard deviation; 95 % CI refers to the 95 % confidence interval

^a^Kruskal Wallis one way analysis of variance was used for this test for significance

In keeping with these findings, evaluation of the impact of HH related comorbidities on utility found all comorbidities were related to lower mean utility than reported for participants reporting no comorbidities (0.76) and the entire HH cohort (0.66) (Table 6). Using the sample mean utility value as the reference case (0.66), participants self-reporting arthritis related to HH had a lower mean utility (0.52: F(1,198) = 10.854, *p* = 0.001). Whilst lower mean utility values were reported for fibrosis, cirrhosis, heart failure, cardiomyopathy, diabetes and porphyria cutanea tarda, only small numbers of participants reported these co-morbidities, therefore these should be interpreted with caution (Table 6).

**Table 6:** Mean utility values by self-reported HH-related comorbidities

HH-related comorbidities^a^	mean HSUV	SD	n
All participants			
no comorbidity	0.76	0.21	100
arthritis	0.52	0.25	35
fibrosis	0.53	0.29	7
cirrhosis	0.61	0.31	5
heart failure	0.58	0.24	3
cardiomyopathy	0.30	-	1
diabetes	0.52	0.33	4
porphyria cutanea tarda	0.02	-	1
Males			
no comorbidity	0.76	0.25	39
arthritis	0.59	0.23	15
fibrosis	0.69	0.05	5
cirrhosis	0.74	0.16	3
Females			
no comorbidity	0.76	0.19	61
arthritis	0.48	0.26	20
fibrosis	0.12	0.02	2
cirrhosis	0.42	0.45	2

Note: HSUV refers to health state utility values; SD standard deviation

^a^Participants were asked if they had been diagnosed with these conditions and that they were considered to be related to HH and iron overload. Participants with these conditions, but were unsure if they were related to HH were not included in this analysis

**Fig. 1:**
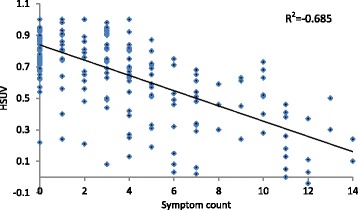
Linear regression of HSUV and symptom count related to HH

Participants were also asked to report on experience of symptoms related to HH and iron overload in the preceding three months (Table 7). Participants were asked if they thought these symptoms were related to HH, possibly related or not related. Only participants reporting their symptoms to be related to HH were included to minimise over-reporting. Of a maximum of 20 symptoms and conditions, the median number experienced by the sample was 3 (SD = 3.8, range 0–15). When compared with the reference HSUV, all symptoms were associated with lower utility. A Pearson correlation coefficient was calculated to assess the relationship between symptom count and HSUV, and a strong negative correlation was found (r = −0.685; *p* < 0.001) (Fig. 1).

**Table 7:** Mean utility values for HH related symptoms

Experienced in the last 3 months	mean HSUV	SD	n	Males			Females		
				mean HSUV	SD	n	mean HSUV	SD	n
General effects									
Chronic fatigue	0.55	0.29	102	0.56	0.31	37	0.55	0.29	65
Weakness	0.49	0.26	87	0.51	0.28	33	0.48	0.26	54
Unexplained weight loss	0.42	0.40	10	1.00	-	1	0.35	0.36	9
Unexplained weight gain	0.50	0.26	30	0.37	0.31	8	0.55	0.23	22
Liver disease									
Abdominal swelling	0.40	0.25	35	0.41	0.33	9	0.39	0.22	26
Abdominal pain/discomfort	0.47	0.26	47	0.51	0.31	12	0.46	0.25	35
Enlarged liver (hepatomegaly)	0.40	0.24	15	0.57	0.19	6	0.29	0.21	9
Cardiac-related problems									
Swelling of feet and/or ankles	0.46	0.23	47	0.43	0.24	17	0.48	0.22	30
Shortness of breath- walking quickly or uphill	0.50	0.27	64	0.54	0.27	24	0.48	0.26	40
Shortness of breath- walking on level ground	0.36	0.26	29	0.39	0.26	14	0.33	0.27	15
Shortness of breath- resting in a chair	0.31	0.25	8	0.32	0.45	3	0.21	0.24	5
Heart failure or weak heart	0.30	-	1	0.30	-	1	-	-	-
Abnormal heart rhythm/ arrhythmia	0.55	0.23	25	0.61	0.17	8	0.52	0.26	17
Heart disease	0.52	0.27	6	0.49	0.28	5	0.71	-	1
Arthritis									
Swollen/tender metacarpophalangeal joints (fingers/hands)	0.48	0.25	58	0.47	0.30	21	0.49	0.22	37
Other joint stiffness/pain/ache	0.55	0.26	96	0.6	0.26	39	0.51	0.24	57
Skin changes									
Change in skin colour	0.45	0.29	25	0.50	0.35	8	0.43	0.27	17
Increased facial hair growth	0.32	0.21	14	-	-	-	0.32	0.21	14
Reproductive									
Loss of libido and/or erectile dysfunction	0.49	0.27	49	0.48	0.27	17	0.49	0.28	32
Unexplained confusion and/or memory loss	0.40	0.24	53	0.39	0.25	18	0.41	0.24	35

Note: HSUV refers to health state utility values; SD standard deviation

## Discussion

This is the first study that reports HSUV measured directly from a cohort with HH. This is of importance, as a lack of robust health economic data has been cited as a barrier to implementing population screening programs for HH [25, 30, 31, 42]. The utility values calculated in this study provide robust estimates that can be used in future economic models of screening interventions. Whilst the sampling strategy may have introduced bias, this has been mitigated by reporting utility values for categories of HH rather than across the study population in general. These values can then be used in combination with penetrance rates in economic models for HH interventions.

Symptomatic stages of HH (categories three and four [25]) were associated with lower utility than asymptomatic stages. The values for all four categories are useful, as they incorporate all aspects of HH and related conditions and can be used to populate health economic models. Previous CUA models have only incorporated specific comorbidities which are associated with significant morbidity and mortality: cirrhosis, diabetes and heart failure, with no consideration of common comorbidities such as arthritis, or symptoms such as fatigue. This may be related to the relatively high prevalence of both fatigue and arthritis amongst other populations, and the difficulties surrounding the aetiologies of both, however there is evidence suggesting that the prevalence of both is higher amongst some groups of HH patients. The prevalence of fatigue amongst general practice patients has been estimated to be between 1.4 and 7.0 % of encounters [43–46]. Work by Allen and colleagues has reported a much higher rate of 22 % for C282Y homozygotes with elevated serum ferritin levels (greater than 1,000 μg/l) [10]. Similarly, arthritis, specifically osteoarthritis, is prevalent in Australia, with 9 % reporting this condition [47]. Allen and colleagues reported use of arthritis medication as a proxy measure for arthritis, noting that 20 % of C282Y homozygotes with serum ferritin greater than 1,000 μg/l reported use of these medications. In combination, these data guided the decision to include arthritis and fatigue in the current study.

To date, just four cost utility analyses have been published on HH screening programs, none of which cited the sources of the utility values employed [7, 48–50]. Values were assigned for cirrhosis, diabetes and heart failure, and in some cases, combinations of these. In a Norwegian study [48], a basal utility value of 1.00 was assumed for all HH conditions except cirrhosis, which was assigned a utility of 0.95, values that are substantially higher than those reported here. Two Canadian studies, by the same research group, used utilities of 0.8 for cirrhosis, 0.9 for diabetes, 0.5 for heart failure, 0.72 for cirrhosis and diabetes, 0.78 for cirrhosis and heart failure, 0.87 for diabetes and heart failure and 0.70 for a combination of cirrhosis, diabetes and heart failure [7, 49]. A fourth study did not provide the utility values used in the modelling [50]. Some concerns arise in respect of these estimates. First, in comparing these utility values to US population normative data, a disparity appears: the mean utility derived from the SF-6D ranged from 0.79 to 0.81 for persons aged 35 to 74, and similarly, using the EQ-5D, mean utility ranged between 0.87 and 0.89 [51]. The fact that the utility estimates that were used in cost utility analyses for participants with health conditions such as cirrhosis and diabetes are similar to or higher than those reported for the general US population indicates these estimates may be incorrect. The likely overestimates of HSUV for HH-related conditions are likely to lead to underestimates of potential utility gains associated with screening interventions.

Second, disease specific HSUV used in these cost utility analyses are also higher than suggested in published literature. A meta-analysis of utility values for liver diseases using a range of approaches to measure utility reported a mean of 0.75 for compensated cirrhosis (range 0.65–0.90) and 0.67 for decompensated cirrhosis (range 0.57–0.81) [52]. Whilst our study did not differentiate cirrhosis in this manner, amongst the small number of participants reporting this condition (*n* = 5), the mean utility (0.61) was slightly lower than reported for decompensated cirrhosis but within the range reported. In contrast, the published cost utility analyses used values of 0.95 [48] and 0.8 [7, 49], higher than the mean values reported for both compensated and decompensated cirrhosis [52]. Similarly, a meta-analysis of utility values for diabetes reported a mean of 0.76 (range 0.53–0.88) [53]. In our study, a mean of 0.52 was reported (*n* = 4), slightly lower than the lower range reported in this meta-analysis. In the three HH cost utility analyses, one used a utility value for diabetes of 1.00 [48], and two used a value of 0.9 [7, 49], both notably higher than published estimates.

Mean utility for heart failure varies depending on the severity of the condition. From a large, multi-site trial that used the EQ-5D, mean utility for different levels of severity based on the New York Heart Association (NYHA) classifications were: class I: 0.815, class II: 0.720, class III: 0.590, class IV: 0.508 [54]. Our study reported a mean of 0.58, however data were available for only three participants, and all were in different NYHA classes. The two Canadian CUA models used a utility value of 0.5 [7, 49], which is similar to the NYHA class 4. In contrast, the Norwegian study assumed a utility of 1.00, which is not in keeping with estimates in the current literature [48].

To date, no economic analysis has incorporated HSUV related to arthritis. This is surprising as arthritis related to iron overload is commonly reported amongst patients diagnosed with HH [10, 55–57]. Whilst HRQoL is not synonymous with HSUV, it can serve as an indicator. A study examining the effects of a range of HH-related comorbidities using the SF-36 found that, compared to cirrhosis and diabetes, arthritis was the single strongest factor that impacted on HRQoL [58]. Whilst the paper was published in 1996, no subsequent studies have incorporated utility values for arthritis. Hawthorne and colleagues, using the AQOL-4D, reported the Australian normative utility value for arthritis as 0.69 (SD 0.26). Our study reported a lower mean value of 0.52 (SD 0.25, *n* = 35). In the current study, both self-reported diagnosis of arthritis related to HH and symptoms suggestive of arthritis were associated with lower mean utility than the sample mean (0.52, 0.48, 0.66 respectively).

Limitations of this study include cross-sectional design and use of convenience sampling. Convenience sampling, which was used as a result of available resourcing, may limit the generalizability of these results. Further, the majority of the respondents were female, despite higher penetrance amongst males. To minimise sampling bias, we have focused on utility values for categories of disease and symptomatology for males and females separately. Whilst an overall sample mean HSUV is likely to be affected by under- or over-reporting from participants with more health problems, the mean values for each category are not affected. This allows for these values, in combination with penetrance estimates from robust epidemiological studies, to be used in HH health economic models.

A further limitation of this study was the reliance on participants’ self-report regarding experience of HH related comorbidities and symptoms. Whilst participants were asked if the comorbidities were related to HH, even with clinical verification, it is difficult to be certain of the aetiology of these. Whilst it can be argued that there may be some over-reporting of symptoms and comorbidities believed to be caused by HH, to minimise this possibility, cases in which participants were unsure of the aetiology have been excluded. Symptoms and comorbidities were only included when participants stated that they were related to HH. Lastly, the small number of participants reporting HH-related comorbidities was also a limitation. Whilst utility values were calculated wherever possible, the small number of respondents means that these data should be interpreted with caution and that no meaningful comparisons can be made between these comorbidities.

## Conclusions

In conclusion, this is the first study to report utility values measured directly from people with HH. Despite study limitations, these values are the best available to date, and can be used to populate health economic models investigating the cost utility of HH interventions.

## References

[1] Worwood M (1999). Inborn errors of metabolism: iron. Br Med Bull.

[2] Wood MJ, Skoien R, Powell LW (2009). The global burden of iron overload. Hepatol Int.

[3] Feder JN (1999). The hereditary hemochromatosis gene (HFE): a MHC class I-like gene that functions in the regulation of iron homeostasis. Immunol Res.

[4] Altes A (2004). Prevalence of the C282Y, H63D, and S65C mutations of the HFE gene in 1,146 newborns from a region of Northern Spain. Genet Test.

[5] Pedersen P, Melsen GV, Milman N (2008). Frequencies of the haemochromatosis gene (HFE) variants C282Y, H63D and S65C in 6,020 ethnic Danish men. Ann Hematol.

[6] Gagne G (2007). Hereditary hemochromatosis screening: effect of mutation penetrance and prevalence on cost-effectiveness of testing algorithms. Clin Genet.

[7] Adams PC, Valberg LS (1999). Screening blood donors for hereditary hemochromatosis: decision analysis model comparing genotyping to phenotyping. Am J Gastroenterol.

[8] Distante S (2004). The origin and spread of the HFE-C282Y haemochromatosis mutation. Hum Genet.

[9] Olynyk JK (1999). A population-based study of the clinical expression of the hemochromatosis gene. N Engl J Med.

[10] Allen KJ (2008). Iron-overload-related disease in HFE hereditary hemochromatosis. N Engl J Med.

[11] Mclaren CE (1995). Prevalence of Heterozygotes for Hemochromatosis in the White-Population of the United-States. Blood.

[12] Adams PC (2005). Hemochromatosis and iron-overload screening in a racially diverse population. N Engl J Med.

[13] Merryweather-Clarke A (2000). Geography of HFE C282Y and H63D mutations. Genet Test.

[14] Biotechnology Australia, A.G.A.B. Australia (2007). Genetics in Family Medicine: The Australian Handbook for General Practitioners: Hereditary haemochromatosis.

[15] Barton J, Edwards CQ (2000). Hemochromatosis: Genetics, patholphysiology, diagnosis and treatment.

[16] Adams P, Brissot P, Powell LW (2000). EASL International Consensus Conference on Haemochromatosis. J Hepatol.

[17] Whitlock EP (2006). Screening for hereditary hemochromatosis: A systematic review for the US Preventive Services Task Force. Ann Intern Med.

[18] Powell LW (2006). Screening for hemochromatosis in asymptomatic subjects with or without a family history. Arch Intern Med.

[19] Asberg A (2001). Screening for hemochromatosis: High prevalence and low morbidity in an unselected population of 65,238 persons. Scand J Gastroenterol.

[20] Ryan E (2002). Underdiagnosis of hereditary haemochromatosis: lack of presentation or penetration?. Gut.

[21] Ajioka RS, Kushner JP (2003). Clinical consequences of iron overload in hemochromatosis homozygotes. Blood.

[22] Adams PC (1997). The relationship between iron overload, clinical symptoms, and age in 410 patients with genetic hemochromatosis. Hepatology.

[23] Allen KJ, Warner B, Delatycki MB (2002). Clinical haemochromatosis in HFE mutation carriers. Lancet.

[24] Mundy L, Merlin T (2003). Population genetic screening for haemochromatosis: identifying asymptomatic “at risk” homozygous individuals. Horizon Scanning Prioritising Summary.

[25] Adams P, Brissot P, Powell L (2000). EASL International Consensus Conference on Haemochromatosis - Part II. Expert document. J Hepatol.

[26] Allen KJ (2008). Population genetic screening for hereditary haemochromatosis: are we a step closer?. Med J Aust.

[27] Barton JC, Acton RT (2000). Population screening for hemochromatosis: has the time finally come?. Curr Gastroenterol Rep.

[28] Gertig DM, Hopper JL, Allen KJ (2003). Population genetic screening for hereditary haemochromatosis. Med J Aust.

[29] Wilson JM, Jungner YG (1968). Principles and practice of mass screening for disease. Bol Oficina Sanit Panam.

[30] Nisselle AE (2004). Implementation of HaemScreen, a workplace-based genetic screening program for hemochromatosis. Clin Genet.

[31] Grosse SD (2010). Population screening for genetic disorders in the 21st century: evidence, economics, and ethics. Public Health Genomics.

[32] Rogowski WH (2009). The Cost-Effectiveness of Screening for Hereditary Hemochromatosis in Germany: A Remodeling Study. Med Decis Making.

[33] de Graaff B (2015). A Systematic review and narrative synthesis of health economic studies conducted for hereditary haemochromatosis. Appl Health Econ Health Policy.

[34] National Institute for Health and Care Excellence (NICE). Guide to the methods of technology appraisal. United Kingdom: NICE; 2013. 27905712

[35] Pharmaceutical Benefits Advisory Committee. Guidelines for preparing submissions to the Pharmaceutical Benefits Advisory Committee. Canberra: Australian Government Department of Health: 2013.

[36] Richardson J, Hawthore G (2001). Negative Utility Scores and Evaluating the AQoL All Worst Health State.

[37] Patrick DL (1994). Measuring preferences for health states worse than death. Med Decis Making.

[38] Hawthorne G, Richardson J, Osborne R (1999). The Assessment of Quality of Life (AQoL) instrument: a psychometric measure of health-related quality of life. Qual Life Res.

[39] Hawthorne G, Richardson J, Day NA (2001). A comparison of the Assessment of Quality of Life (AQoL) with four other generic utility instruments. Ann Med.

[40] Hawthorne G, Korn S, Richardson J (2013). Population norms for the AQoL derived from the 2007 Australian National Survey of Mental Health and Wellbeing. Aust N Z J Public Health.

[41] Centre for Health Economics, Monash University. *AQoL.* Assessment of Quality of Life. 2014 [cited 2014 10/11/2014]; Available from: http://www.aqol.com.au/index.php/scoring-algorithms?id=82.

[42] Mundy L, Merlin T, AHTA (2004). Population genetic screening for haemochromatosis: identifying asymptomatic “at risk” homozygous individuals. Horizon Scanning Prioritising Summary.

[43] Britt H, Henderson J, Charles J (2012). General practice activity in Australia 2011–12. General Practice Series.

[44] Gallagher AM (2004). Incidence of fatigue symptoms and diagnoses presenting in UK primary care from 1990 to 2001. J R Soc Med.

[45] Cullen W, Kearney Y, Bury G (2002). Prevalence of fatigue in general practice. Ir J Med Sci.

[46] Kenter EG (2003). Tiredness in Dutch family practice. Data on patients complaining of and/or diagnosed with “tiredness”. Fam Pract.

[47] Australian Bureau of Statistics. National Health Survey: First Results, 2014–15. 2015 5.1.2016]; Available from: http://www.abs.gov.au/ausstats/abs@.nsf/Lookup/by%20Subject/4364.0.55.001~2014-15~Main%20Features~Arthritis%20and%20osteoporosis~8.

[48] Asberg A (2002). Benefit of population-based screening for phenotypic hemochromatosis in young men. Scand J Gastroenterol.

[49] Adams PC, Kertesz AE, Valberg LS (1995). Screening for hemochromatosis in children of homozygotes: prevalence and cost-effectiveness. Hepatology (Baltimore, Md.).

[50] Adams PC, Gregor JC, Kertesz AE, Valberg LS (1995). Screening blood donors for hereditary hemochromatosis: decision analysis model based on a 30-year database. Gastroenterol Clin North Am..

[51] Fryback DG (2007). US norms for six generic health-related quality-of-life indexes from the National Health Measurement study. Med Care.

[52] McLernon DJ, Dillon J, Donnan PT (2008). Health-state utilities in liver disease: a systematic review. Med Decis Making.

[53] Lung TW (2011). A meta-analysis of health state valuations for people with diabetes: explaining the variation across methods and implications for economic evaluation. Qual Life Res.

[54] Holland R (2010). Patients’ self-assessed functional status in heart failure by New York Heart Association class: a prognostic predictor of hospitalizations, quality of life and death. J Card Fail.

[55] Sahinbegovic E (2010). Musculoskeletal disease burden of hereditary hemochromatosis. Arthritis Rheum.

[56] Barton JC (2005). Initial screening transferrin saturation values, serum ferritin concentrations, and HFE genotypes in whites and blacks in the Hemochromatosis and Iron Overload Screening Study. Genet Test.

[57] McDonnell SM (1999). A survey of 2,851 patients with hemochromatosis: Symptoms and response to treatment. Am J Med.

[58] Adams PC, Speechley M (1996). The effect of arthritis on the quality of life in hereditary hemochromatosis. J Rheumatol.

